# Antidiabetic Potential of Five Flower Remedy: A Thai Traditional Remedy With α-Amylase and α-Glucosidase Inhibition, Synergistic Interactions, and Mechanistic Insights

**DOI:** 10.1155/sci5/5516791

**Published:** 2025-09-19

**Authors:** Piriya Chonsut, Prapaporn Chaniad, Thanchanok Limcharoen, Ichwan Ridwan Rais, Paratthakorn Sangkaew

**Affiliations:** ^1^Department of Applied Thai Traditional Medicine, School of Medicine, Walailak University, Nakhon Si Thammarat 80160, Thailand; ^2^Center of Excellence in Tropical Pathobiology, Walailak University, Nakhon Si Thammarat 80160, Thailand; ^3^Department of Medical Sciences, School of Medicine, Walailak University, Nakhon Si Thammarat 80160, Thailand; ^4^Department of Pharmaceutical Sciences and Technology Program, Faculty of Pharmaceutical Sciences, Chulalongkorn University, Bangkok 10330, Thailand; ^5^Department of Pharmaceutical Biology, Faculty of Pharmacy, Universitas Ahmad Dahlan, Yogyakarta 55164, Indonesia

## Abstract

Five Flower Remedy (FFR), a Thai traditional medicine, has a history of use for various ailments. This study investigated its antidiabetic potential by examining its impact on key carbohydrate-metabolizing enzymes, α-amylase, and α-glucosidase. We explored FFR's inhibitory effects, synergistic interactions with the standard antidiabetic drug acarbose, and the underlying mechanisms involved. *In vitro* analysis revealed that FFRA significantly inhibited both α-amylase and α-glucosidase. Importantly, a synergistic effect was observed when FFRA extract was combined with acarbose, particularly at low concentrations for α-amylase and α-glucosidase inhibition. This suggests a potential benefit in combination therapy. Furthermore, FFRE demonstrated greater inhibitory potency against both enzymes compared to acarbose. Mechanistically, FFRE inhibited α-amylase through competitive inhibition and α-glucosidase through noncompetitive inhibition, indicating distinct modes of action. These findings provide scientific validation for the traditional use of FFR in managing diabetes and offer valuable insights into its pharmacological properties. The observed synergistic effects and distinct inhibition mechanisms highlight FFR's potential as a therapeutic agent. This study serves as a foundation for future research, including identifying the specific bioactive components responsible for FFR's antidiabetic effects, *in vivo* studies to confirm these findings in living organisms, and ultimately, clinical trials to evaluate its efficacy and safety in human subjects. Further investigation could pave the way for developing FFR as a novel therapeutic approach for diabetes management.

## 1. Introduction

Diabetes mellitus (DM), a chronic metabolic disorder characterized by high blood glucose levels, has become a major global health concern [1]. Type 2 diabetes, in particular, is closely linked to insulin resistance and impaired glucose metabolism [2]. Current pharmacological treatments for diabetes mainly focus on managing blood glucose levels through the inhibition of enzymes such as α-amylase and α-glucosidase, which are key players in carbohydrate digestion [3]. Despite the availability of conventional drugs, concerns over side effects and long-term efficacy have prompted a search for alternative therapies [4]. Traditional remedies, especially those with known bioactive properties, offer promising solutions for the management of diabetes, while also addressing these concerns [5, 6].

One such traditional remedy is the Five Flower Remedy (FFR), a Thai herbal preparation that has been used for centuries for various health conditions [7]. FFR, a traditional medicine, comprises extracts from five distinct plants: *Jasminum sambac* Ait. (JS), *Mimusops elengi* L. (ME), *Mesua ferrea* L. (MF), *Mammea siamensis* Kosterm (MS), and *Nelumbo nucifera* Gaertn. (NC) [8]. While each of these plants contributes unique bioactive compounds believed to confer therapeutic benefits [7], the antidiabetic potential of FFR, particularly its capacity to inhibit α-amylase and α-glucosidase, remains largely unexplored. This lack of extensive research makes FFR an intriguing candidate for further investigation in diabetes management.

Enzyme inhibition is a central strategy in the treatment of diabetes, with α-amylase and α-glucosidase inhibitors playing a significant role in reducing postprandial blood glucose spikes [9]. By delaying the breakdown of carbohydrates into glucose, these inhibitors help lower the rate at which glucose is absorbed into the bloodstream [10]. In this study, we investigate the antidiabetic potential of FFR by evaluating its ability to inhibit both α-amylase and α-glucosidase and explore the synergistic effects that may arise from combining various plant extracts within the remedy. This approach not only evaluates the individual efficacy of the extracts but also examines how they might interact to enhance therapeutic outcomes.

In addition to enzyme inhibition, understanding the mechanistic insights behind the interactions between the components of FFR is crucial. Synergistic interactions between herbal extracts may provide enhanced therapeutic benefits compared to single-agent treatments [11]. This study employs a combination index (CI) analysis to assess the degree of synergy or antagonism between the FFR extracts and acarbose, a commonly used pharmaceutical α-amylase and α-glucosidase inhibitor [12]. By analyzing these interactions, we aim to identify optimal doses and therapeutic combinations that may reduce the required dosages of conventional drugs while enhancing overall efficacy.

The ultimate goal of this research is to provide a deeper understanding of the antidiabetic mechanisms of FFR, with a particular focus on its effects on carbohydrate digestion and glucose regulation. The findings from this study may pave the way for the development of novel, complementary therapies that combine the benefits of traditional herbal remedies with modern pharmacological treatments, offering a holistic approach to diabetes management.

## 2. Materials and Methods

### 2.1. Materials

Thai Oil Co., Ltd., a Thailand-based company, supplied all the necessary solvents for the extraction processes. The α-amylase and α-glucosidase enzymes (from *Saccharomyces cerevisiae*), 2-chloro-4-nitrophenyl-α-D-maltotrioside (*CNPG3*), *p*-nitrophenol-α-glucopyranoside, acarbose, bovine serum albumin, sodium azide, sodium carbonate, and dimethyl sulfoxide (DMSO) were obtained from Sigma, Sigma-Aldrich (Germany). Phosphate-buffered saline (PBS) was provided by Gibco® (Life Technologies, Paisley, Scotland).

### 2.2. Plant Material and Management

The plant samples were sourced from Sai Buri Herb Shop in Hat Yai District, Songkhla Province, with a total of five samples, each weighing 300 g. Upon receipt, the quality was evaluated by weighing the samples and removing any impurities. The plant species included in the study were *Jasminum sambac* Ait., *Mimusops elengi* L., *Mesua ferrea* Linn., *Mammea siamensis* Kosterm., and *Nelumbo nucifera* Gaertn. The authenticity of these plants was confirmed by a certified Thai traditional pharmacist. As part of the reference collection, samples of both the remedies and individual ingredients were collected and preserved in the authors' herbarium at the Department of Applied Thai Traditional Medicine, Walailak University, Nakhon Si Thammarat, Thailand (Table 1).

### 2.3. Preparation of Extraction

Each herbal sample and FFR, weighing 60 g each, were extracted using two distinct methods. The plant components were mixed in equal proportions (12 g each) in a 1:1:1:1:1 ratio. Method 1: The samples were placed in bottles and fully submerged in 95% ethanol, ensuring complete coverage by the solvent. The bottles were sealed and left to macerate for 7 days. Afterward, the samples were transferred to a temperature-controlled shaking incubator. Following maceration, the solvent was filtered using a Buchner funnel. The residue underwent three additional rounds of maceration using the same process. Method 2: The samples were immersed in distilled water and heated on a hot plate set to 80°C. The first extraction lasted 30 min, followed by two shorter extractions of 15 min each. The resulting aqueous extract was concentrated with a rotary evaporator until its volume was reduced to about one-third of the original. For both methods, the aqueous extract was dried using a vacuum freeze dryer (lyophilizer) to yield a powdered extract, which was weighed and documented. The ethanolic extract was further concentrated using a rotary evaporator until 25–30 mL remained. This concentrate was processed in a vacuum centrifuge concentrator to achieve a thick, sticky solution, which was also weighed and recorded. Finally, the dried plant extracts were stored in screw-cap containers at 4°C for future use [13].

### 2.4. α-Amylase Inhibitory Assay

A modified approach based on a previous study was employed to evaluate the α-amylase inhibitory activity of the FFR [14]. In this assay, α-amylase enzymatically hydrolyzes *CNPG3*, generating glucose and 2-chloro-4-nitrophenol (*CNP*), which imparts a yellow coloration to the solution and absorbs light at 405 nm. To initiate the reaction, a standardized solution or test sample (starting concentration of 8 mg/mL) was mixed with 20 μL of 50 mM sodium phosphate buffer (pH 6.8). Subsequently, 20 μL of α-amylase solution (300.0 U/mL) prepared in the same buffer was added to each well of a 96-well plate and incubated at room temperature for 10 min. After incubation, 20 μL of 2.0 mM *CNPG3* was introduced, mixed thoroughly, and further incubated for 10 min at room temperature. The reaction was then terminated by adding 40 μL of 1.0 mM sodium carbonate (Na_2_CO_3_) solution. Absorbance was recorded at 405 nm using a microplate reader to quantify enzymatic activity. The percentage of α-amylase inhibition was calculated using the following formula:

(1) $$\begin{array}{c} \%\text{ Inhibition}=\left[\frac{\left({A405}^{\text{Control}}-{A405}^{\text{Treatment}}\right)}{{A405}^{\text{Control}}}\right]\times 100, \end{array}$$

where A405^Control^ represents the absorbance at 405 nm in the control sample (without extract) and A405^Treatment^ represents the absorbance at 405 nm in the treated sample (with extract).

### 2.5. α-Glucosidase Inhibitory Assay

The α-glucosidase inhibitory activity of FFR and its plant extracts was evaluated using the method outlined by a previous study [15]. In summary, 50 μL of plant extract at a concentration of 2 mg/mL was mixed with 50 μL of α-glucosidase (1 U/mL) derived from *S. cerevisiae* (Type I, lyophilized powder, Sigma, EC 3.2.1.20) and incubated at 37°C for 2 min. The reaction mixture also included 50 μL of 0.1 M phosphate buffer (pH 7.0). The reaction was initiated by adding 50 μL of *p*-nitrophenyl-α-D-glucopyranoside (*pNPG*) as the substrate. The formation of *p*-nitrophenol (*pNP*) was monitored at 405 nm every 30 s for 10 min using a microplate reader. Absorbance values from the test wells were adjusted by subtracting the blank absorbance before calculating the reaction velocity using equation (2). The initial velocity (*V*) of each sample was determined, and the percentage of enzyme inhibition was computed using equation (3). The IC_50_ value was obtained from a calibration curve that plotted the percentage of inhibition against five sample concentrations, ranging from 125 to 2000 μg/mL. All experiments were performed in triplicate.

(2) $$\begin{array}{c} \text{Velocity}=\frac{\Delta \text{Absorbance at }405\text{ nm}}{\Delta \text{Time}}, \end{array}$$

(3) $$\begin{array}{c} \%\text{ Inhibition}=\left[\frac{\left({A405}^{\text{Control}}-{A405}^{\text{Treatment}}\right)}{{A405}^{\text{Control}}}\right]\times 100, \end{array}$$

where A405^Control^ is the absorbance at 405 nm in the control sample without the extract and A405^Treatment^ is the absorbance at 405 nm after treatment with the extract.

### 2.6. The Synergistic Inhibitory Effect of FFR and Its Plant Ingredient Extracts Combined With Acarbose on the α-Amylase Inhibitory Assay

The study used CompuSyn Software from ComboSyn, Inc. (Paramus, NJ, USA) (https://www.combosyn.com/, accessed on January 21, 2025) to analyze the combined effects of various plant extracts with the potential to inhibit the α-amylase enzyme, along with the standard acarbose. The Chou–Talalay method for evaluating drug combinations, as described in a previous study [16], was employed. The results, including the plot of fraction affected (Fa) versus CI and the normalized isobologram, were generated through automated computer simulation. In this context, a CI value of less than 1 indicated synergistic, a CI of 1 signified an additive effect, and a CI greater than 1 represented antagonistic [16, 17]. The study's primary goal was to explore effective and safe approaches for managing diabetes, including the potential development of a novel Thai herbal remedy.

### 2.7. The Synergistic Inhibitory Effect of FFR and Its Plant Ingredient Extracts Combined With Acarbose on the α-Glucosidase Inhibitory Assay

To construct dose–response curves, IC_50_ values of acarbose and three concentrations of FFR and its plant ingredients (ranging from 0.5 to 2IC_50_) were utilized on α-glucosidase enzyme, following the Chou and Talalay methodology [16]. The CI values were calculated using CompuSyn software (Version 1.0; https://www.combosyn.com/), accessed on January 15, 2025, which was essential for evaluating the interaction between the compounds. Based on the CI values, combinations were classified as synergistic (CI < 1), additive (CI = 1), or antagonistic (CI > 1) [16, 17].

### 2.8. Enzyme Kinetic Determination of α-Amylase Inhibitory Assay

The mode of action and inhibition constant (Ki) for the α-amylase inhibitory effect was determined based on a previously established method [18], with slight modifications to the enzymatic reaction. The Lineweaver–Burk equation and Dixon plot were utilized for kinetic analysis. Three concentrations of active crude extracts (31.25–500 μg/mL) were selected based on their inhibitory activity. The assay was conducted using six different substrate concentrations (0.1–5 mM). A fixed amount of α-amylase was incubated with varying concentrations of *CNPG3* at 37°C for 15 min, both in the presence and absence of test samples at concentrations equivalent to their IC_50_ values. The inhibition kinetics were then analyzed using the appropriate equations (equations (4) and (5)).

Lineweaver–Burk equation is as follows:

(4) $$\begin{array}{c} \frac{1}{V}=\frac{K_{m}}{V_{\max}}\left(\frac{1}{S}\right)+\frac{1}{V_{\max}}, \end{array}$$

Dixon equation is as follows:

(5) $$\begin{array}{c} \frac{K_{m}}{V_{\max}}=\frac{K_{m}}{V_{\max}}\left(1+\frac{I}{K_{i}}\right). \end{array}$$

### 2.9. Enzyme Kinetic Determination of α-Glucosidase Inhibitory Assay

The mode of inhibition was determined using the double reciprocal Lineweaver–Burk plot, while the inhibition constants (*K*_*i*_) were obtained from secondary plots [19]. For competitive inhibition, the *K*_*i*_ value represents the inhibitor binding to the free enzyme, whereas for uncompetitive inhibition, the *K*_*i*_ value indicates binding to the enzyme–substrate complex. The enzyme inhibition assay was conducted as described earlier. To evaluate the inhibition mechanism, six concentrations of *p*-nitrophenyl-α-D-glucopyranoside (*pNPG*) (0.1–5 mM) were used in combination with a fixed enzyme concentration (1 unit/mL) and three different concentrations of each active sample. The inhibition kinetics were then analyzed using the previous equations.

### 2.10. Statistical Analysis

In this study, we calculated the average values from three independent experiments and presented them as the mean ± standard deviation (SD). For all analyses, we employed one-way analysis of variance (ANOVA) using GraphPad Prism software version 8.0. Statistical significance was determined if the *p* value was below 0.05, indicating a 95% confidence level.

## 3. Results

### 3.1. Percentage Yield

Table 2 presents the extraction yields (% w/w) of ethanolic and aqueous extracts from the medicinal plants comprising FFR. Aqueous extraction generally yielded more extract than ethanolic extraction across most species. Specifically, the highest aqueous yields were observed for MS (24.36%), followed by ME (21.24%) and MF (15.87%). Conversely, FFR itself showed the highest ethanolic yield (17.78%), followed by ME (14.54%) and MS (11.47%). JS notably yielded less with ethanolic extraction (2.85%) compared to aqueous extraction (7.45%). The higher aqueous yields for ME and MF suggest that their bioactive components are more water-soluble. Similarly, MS and NC also yielded more extract in aqueous medium. However, FFR itself exhibited a greater affinity for ethanol, yielding more extract in the ethanolic extraction (17.78%) than the aqueous extraction (10.72%), suggesting that alcohol is a more effective solvent for extracting its bioactive components.

### 3.2. The α-Amylase Inhibitory Activity of FFR and Its Plant-Derived Ingredients

**Figure 1:**
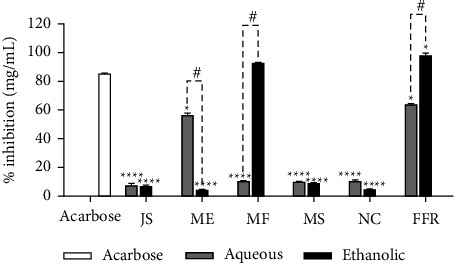
The α-amylase inhibitory activity of all extracts with standard drug acarbose (JS, *J. sambac*; ME, *M. elengi*; MF, *M. ferrea*; MS, *M. siamensis*; NC, *N. nucifera*; FFR, five flower remedy) (statistical significance levels are indicated at ^∗^*p* < 0.01 compared to acarbose (standard drug) ^∗∗∗∗^*p* < 0.0001 compared to acarbose (standard drug) and ^#^*p* < 0.0001 compared to the aqueous and ethanolic extracts).

The α-amylase inhibitory activity of various plant extracts, including both aqueous and ethanolic forms, in comparison to the standard drug acarbose, is illustrated in Figure 1. The *y*-axis represents the percentage of inhibition (mg/mL), while the *x*-axis categorizes the tested plant extracts: JS, ME, MF, MS, NS, and FFR. Acarbose, serving as the standard control and represented by a white bar, exhibits α-amylase inhibitory activity, achieving approximately 85.38 ± 0.18% inhibition. Aqueous extracts are shown as gray bars, whereas ethanolic extracts are depicted as black bars. In general, ethanolic extracts exhibit stronger inhibitory activity than aqueous extracts, indicating a more effective extraction of active compounds in ethanol. JS: Both aqueous and ethanolic extracts exhibit significantly lower inhibition compared to acarbose. The aqueous extract shows minimal inhibition, while the ethanolic extract demonstrates slightly higher activity (*p* < 0.0001). ME: The ethanolic extract exhibits moderate inhibition, which is significantly higher than the aqueous extract (# denotes a significant difference between extract types). MF: The ethanolic extract demonstrates the highest inhibitory effect among all plant extracts, approaching 92.51 ± 0.79% inhibition, like acarbose. The aqueous extract, however, shows minimal inhibition. MS and NC: Both aqueous and ethanolic extracts display low inhibition levels, with statistically significant differences (*p* < 0.0001). For the FFR, the ethanolic extract (FFRE) exhibits strong α-amylase inhibition, nearly reaching 100%, whereas the aqueous extract (FFRA) demonstrates moderate inhibition. The # symbol indicates a significant difference between the aqueous and ethanolic extracts (*p* < 0.05).

### 3.3. The IC_50_ Values for α-Amylase Inhibitory Activity of FFR and Its Plant-Derived Ingredients

**Figure 2:**
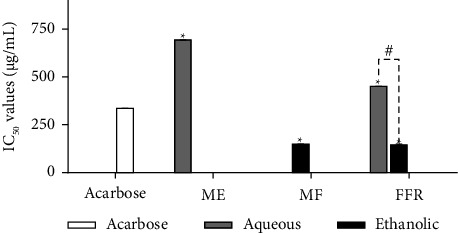
The IC_50_ values for α-amylase inhibitory activity of all extracts with standard drug acarbose (ME, *M. elengi*; MF, *M. ferrea*; FFR, five flower remedy) (statistical significance levels are indicated at ^∗^*p* < 0.0001 compared to acarbose (standard drug) and ^#^*p* < 0.0001 compared to the aqueous and ethanolic extracts).

The α-amylase inhibitory activity of FFR and its fractions is demonstrated in Figure 2, expressed as IC_50_ values (μg/mL). The inhibitory potential of FFR, its constituents extracted using aqueous and ethanolic solvents, and the known inhibitor acarbose were compared. Acarbose, represented by a white bar, demonstrated a low IC_50_ of 338.04 μg/mL, consistent with its established efficacy. The ME extract, obtained using an aqueous solvent, exhibited a higher IC_50_ than acarbose, indicating comparatively weaker inhibition. Conversely, the ethanolic extract of the MF showed a lower IC_50_ (150.24 μg/mL) than acarbose, suggesting improved inhibitory activity after fractionation. FFRE demonstrated the strongest α-amylase inhibitory potency, with specific IC_50_ values (145.05 μg/mL) provided in the figure. Asterisks (∗) above the ME, MF, and FFR extracts indicate statistically significant differences (*p* < 0.0001), presumably compared to acarbose. The # symbol denotes a significant difference between the aqueous and ethanolic FFR extracts. These findings underscore the influence of both fractionation and solvent choice on the α-amylase inhibitory capacity of FFR, with the ethanolic MF and FFR extracts exhibiting the most potent inhibition among the FFR-derived samples tested.

### 3.4. The α-Glucosidase Inhibitory Activity of FFR and Its Plant-Derived Ingredients

**Figure 3:**
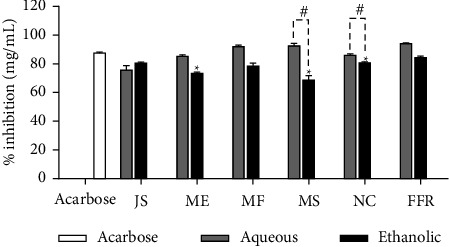
The α-glucosidase inhibitory activity of all extracts with standard drug acarbose (JS, *J. sambac*; ME, *M. elengi*; MF, *M. ferrea*; MS, *M. siamensis*; NC, *N. nucifera*; FFR, five flower remedy) (statistical significance levels are indicated at ^∗^*p* < 0.0001 compared to acarbose (standard drug) and ^#^*p* < 0.0001 compared to the aqueous and ethanolic extracts).

The α-glucosidase inhibitory effects of aqueous and ethanolic extracts from the FFR remedy and its individual plant-derived components (JS, ME, MF, MS, NC, and FFR) were evaluated in comparison to the standard inhibitor acarbose. The results, expressed as percentage inhibition (mg/mL), are presented in Figure 3. A clustered column chart illustrates the inhibitory activity of each extract at a concentration of 2 mg/mL alongside the positive control, acarbose. Acarbose, used as the reference standard, demonstrated a percentage inhibition of 87.90 ± 0.06%, showcasing its strong inhibitory effect on α-glucosidase. Generally, the aqueous extracts exhibited higher inhibitory activity than their ethanolic counterparts. JS extracts: Both aqueous and ethanolic extracts of JS displayed high inhibition, closely matching acarbose's activity, with inhibition rates of 75.75 ± 3.40% and 81.05 ± 0.30%, respectively. ME extracts: The aqueous extract of ME showed slightly lower inhibition than the ethanolic extract. The aqueous form, marked by an asterisk (∗), indicated a statistically significant difference between the two extracts. MF extracts: Both extracts demonstrated strong inhibitory effects with minimal differences, showing 92.41 ± 1.13% for the aqueous extract and 78.61 ± 2.01% for the ethanolic extract. MS extracts: The aqueous extract of MS significantly outperformed the ethanolic extract, achieving 92.41 ± 1.71% inhibition compared to 68.92 ± 3.03%, as indicated by the asterisk (∗) and hash (#) symbols, denoting statistical significance. NC extracts: Similar to MS, the aqueous extract exhibited considerably higher inhibition than the ethanolic extract. FFR extracts: FFRA showed the highest inhibition against α-glucosidase among all tested samples, with an impressive inhibition rate of 94.40 ± 0.55%. Overall, these findings highlight the superior efficacy of aqueous extracts, particularly for MS, NC, and FFR, suggesting that water-based extraction may better preserve or extract potent α-glucosidase inhibitory compounds.

### 3.5. The IC_50_ Values for α-Glucosidase Inhibitory Activity of FFR and Its Plant-Derived Ingredients

**Figure 4:**
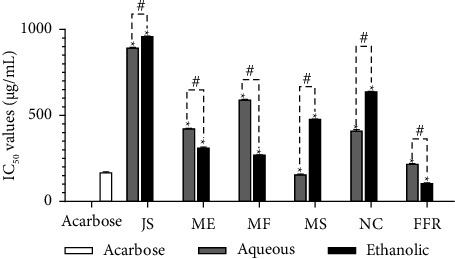
The IC_50_ values for α-glucosidase inhibitory activity of all extracts with standard drug acarbose (JS, *J. sambac*; ME, *M. elengi*; MF, *M. ferrea*; MS, *M. siamensis*; NC, *N. nucifera*; FFR, five flower remedy) (statistical significance levels are indicated at ^∗^*p* < 0.0001 compared to acarbose (standard drug) and ^#^*p* < 0.0001 compared to the aqueous and ethanolic extracts).

Figure 4 presents the α-glucosidase inhibitory activity of FFR and its individual plant components, comparing the IC_50_ values (μg/mL) of both aqueous and ethanolic extracts against the standard inhibitor, acarbose. IC_50_ values reflect the concentration needed to inhibit 50% of α-glucosidase activity, with lower values indicating stronger inhibitory potency. The figure employs a clustered column chart to illustrate the inhibitory effects of each extract alongside acarbose. Statistical significance is denoted by an asterisk (∗) for differences between aqueous and ethanolic extracts and a hash (#) for variations relative to acarbose. Acarbose, the reference inhibitor, displayed a low IC_50_ value, confirming its strong α-glucosidase inhibitory capability. In contrast, both aqueous and ethanolic extracts of JS exhibited high IC_50_ values (893.55 μg/mL and 960.31 μg/mL, respectively), indicating comparatively weaker inhibitory activity. For ME, the ethanolic extract showed a lower IC_50_ value (312.18 μg/mL) compared to its aqueous counterpart, suggesting greater potency. The MF extracts demonstrated moderate inhibitory activity, with the aqueous form exhibiting an IC_50_ of 591.60 μg/mL. In the case of MS, the aqueous extract had a significantly lower IC_50_ value (157.43 μg/mL) than the ethanolic extract, indicating more effective enzyme inhibition. A similar trend was observed with NC, where the aqueous extract displayed substantially stronger inhibitory action compared to the ethanolic form. Notably, FFRE exhibited the lowest IC_50_ value among all tested samples (104.99 μg/mL), highlighting its potent inhibitory potential against α-glucosidase. These findings suggest that the FFRE may serve as a promising natural inhibitor for managing postprandial hyperglycemia.

### 3.6. CI Test of the FFR and Its Plant-Derived Ingredients on α-Amylase Inhibitory Activity

**Figure 5:**
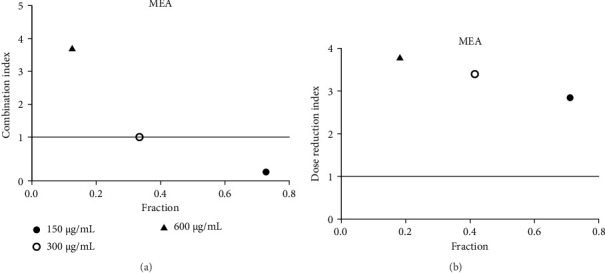
(a) Combination index plot of nonconstant combo acarbose-MEA on α-amylase (MEA, aqueous extract of *M. elengi* combined with acarbose, fraction; the default effect). (b) Dose reduction index of nonconstant combo acarbose-MEA (MEA, aqueous extract of *M. elengi* combined with acarbose, fraction; the default effect).

**Figure 6:**
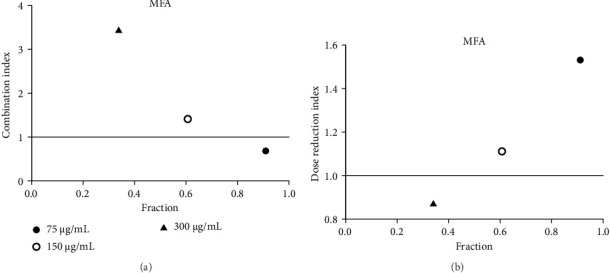
(a) Combination index plot of nonconstant combo acarbose-MFA on α-amylase (MFA, aqueous extract of *M. ferrea* combined with acarbose, fraction; the default effect). (b) Dose reduction index of nonconstant combo acarbose-MFA (MFA, aqueous extract of *M. ferrea* combined with acarbose, fraction; the default effect).

**Figure 7:**
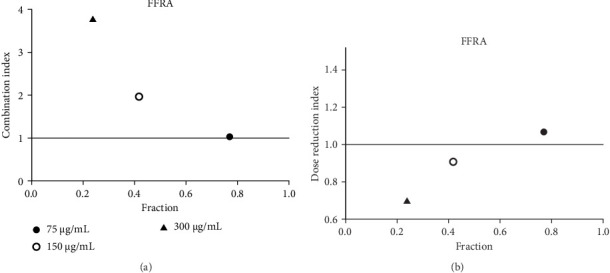
(a) Combination index plot of nonconstant combo acarbose-FFRA on α-amylase (FFRA, aqueous extract of five flower remedy combined with acarbose, fraction; the default effect). (b) Dose reduction index of nonconstant combo acarbose-FFRA (FFRA, aqueous extract of five flower remedy combined with acarbose, fraction; the default effect).

**Figure 8:**
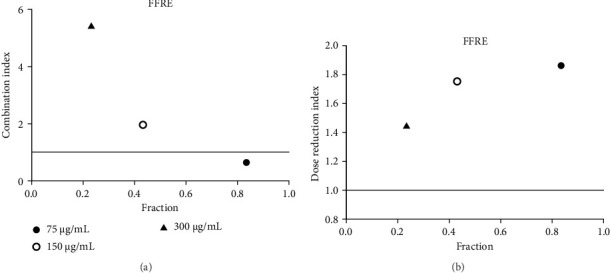
(a) Combination index plot of nonconstant combo acarbose-FFRE on α-amylase (FFRE, ethanolic extract of five flower remedy combined with acarbose, fraction; the default effect). (b) Dose reduction index of nonconstant combo acarbose-FFRE (FFRE, ethanolic extract of five flower remedy combined with acarbose, fraction; the default effect).

Acarbose was tested with four extracts (MEA, MFA, FFRA, FFRE) in a CI analysis. A fixed dose of acarbose (300 μg/mL) was combined with varying extract concentrations (0.5–2 × IC_50_). The CI index values of all samples are shown in Tables 3, 4, 5, and 6. MEA strongly inhibited the enzyme, with effects depending on the dose: synergistic at 150 μg/mL (CI = 0.49), additive at 300 μg/mL (CI = 1.04), and antagonistic at 600 μg/mL (CI = 3.83). MEA at 150–300 μg/mL reduced the acarbose dose needed, while 600 μg/mL provided no benefit (Figure 5(b)). MFA showed synergistic effects only at the lowest dose (75 μg/mL, CI = 0.69) (Table 4 and Figure 6). FFRA had a synergistic effect at 75 μg/mL, additive at 150 μg/mL, and antagonistic at 300 μg/mL. FFRA at 75–150 μg/mL reduced the acarbose dose, but 300 μg/mL was ineffective (Figure 7(b)). FFRE showed synergy at lower doses (75–150 μg/mL) and potential dose reduction benefits. At higher doses (300 μg/mL), the effect diminished, leading to additivity or antagonism (Figure 8). These results highlight the importance of dose selection for maximizing combination therapy benefits.

### 3.7. CI Test of the FFR and Its Plant-Derived Ingredients on α-Glucosidase Inhibitory Activity

**Figure 9:**
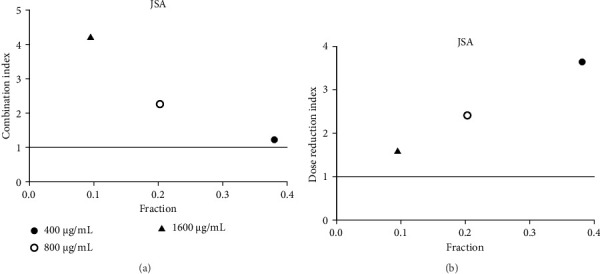
(a) Combination index plot of nonconstant combo acarbose-JSA on α-glucosidase (JSA, aqueous extract of *J. sambac* combined with acarbose, fraction; the default effect). (b) Dose reduction index of nonconstant combo acarbose-JSA (JSA, aqueous extract of *J. sambac* combined with acarbose, fraction; the default effect).

**Figure 10:**
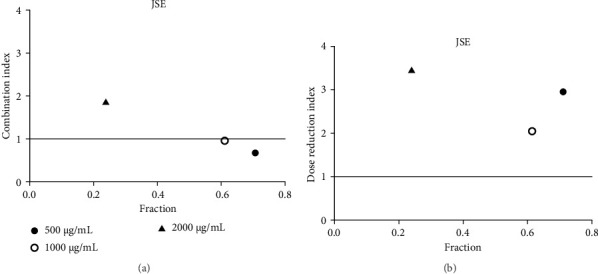
(a) Combination index plot of nonconstant combo acarbose-JSE on α-glucosidase (JSE, ethanolic extract of *J. sambac* combined with acarbose, fraction; the default effect). (b) Dose reduction index of nonconstant combo acarbose-JSE (JSE, ethanolic extract of *J. sambac* combined with acarbose, fraction; the default effect).

**Figure 11:**
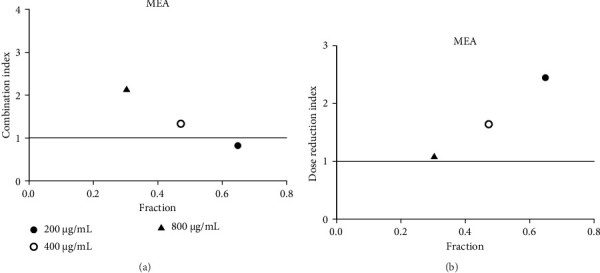
(a) Combination index plot of nonconstant combo acarbose-MEA on α-glucosidase (MEA, aqueous extract of *M. elengi* combined with acarbose, fraction; the default effect). (b) Dose reduction index of nonconstant combo acarbose-MEA (MEA, aqueous extract of *M. elengi* combined with acarbose, fraction; the default effect).

**Figure 12:**
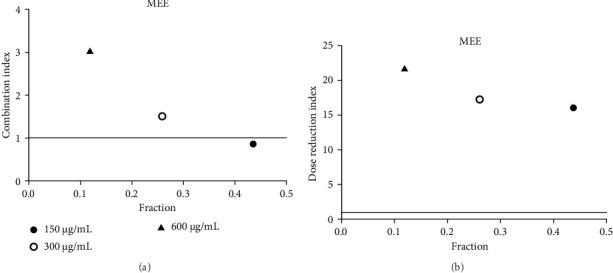
(a) Combination index plot of nonconstant combo acarbose-MEE on α-glucosidase (MEE, ethanolic extract of *M. elengi* combined with acarbose, fraction; the default effect). (b) Dose reduction index of nonconstant combo acarbose-MEE (MEE, ethanolic extract of *M. elengi* combined with acarbose, fraction; the default effect).

**Figure 13:**
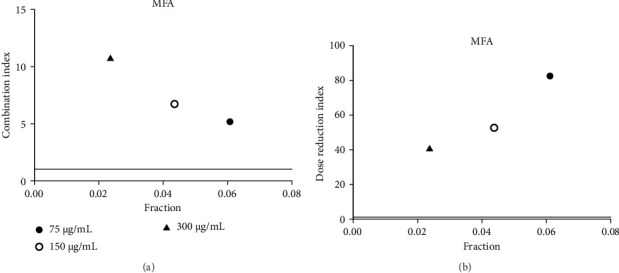
(a) Combination index plot of nonconstant combo acarbose-MFA on α-glucosidase (MFA, aqueous extract of *M. ferrea* combined with acarbose, fraction; the default effect). (b) Dose reduction index of nonconstant combo acarbose-MFA (MFA, aqueous extract of *M. ferrea* combined with acarbose, fraction; the default effect).

**Figure 14:**
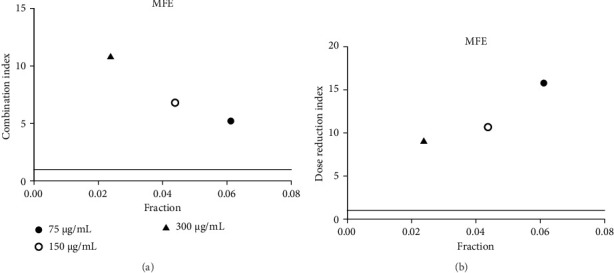
(a) Combination index plot of nonconstant combo acarbose-MFE on α-glucosidase (MFE, ethanolic extract of *M. ferrea* combined with acarbose, fraction; the default effect). (b) Dose reduction index of nonconstant combo acarbose-MFE (MFE, ethanolic extract of *M. ferrea* combined with acarbose, fraction; the default effect).

**Figure 15:**
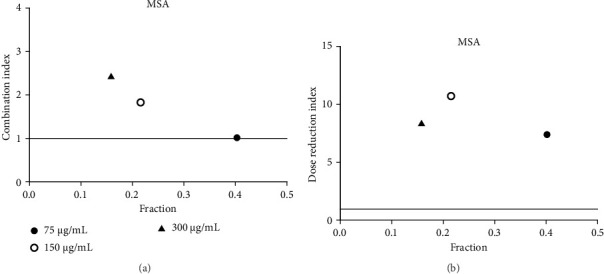
(a) Combination index plot of nonconstant combo acarbose-MSA, on α-glucosidase (MSA, aqueous extract of *M. siamensis* combined with acarbose, fraction; the default effect). (b) Dose reduction index of nonconstant combo acarbose-MSA (MSA, aqueous extract of *M. siamensis* combined with acarbose, fraction; the default effect).

**Figure 16:**
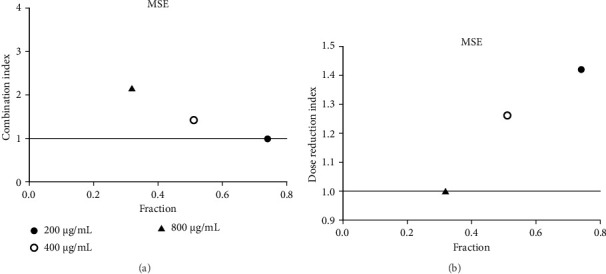
(a) Combination index plot of nonconstant combo acarbose-MSE on α-glucosidase (MSE, ethanolic extract of *M. siamensis* combined with acarbose, fraction; the default effect). (b) Dose reduction index of nonconstant combo acarbose-MSE (MSE, ethanolic extract of *M. siamensis* combined with acarbose, fraction; the default effect).

**Figure 17:**
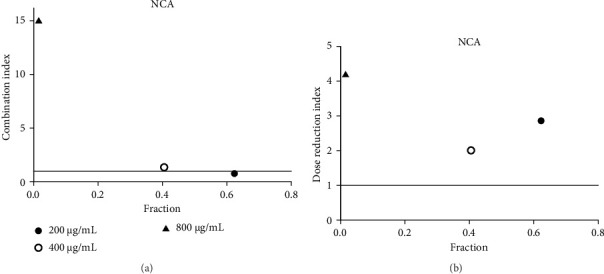
(a) Combination index plot of nonconstant combo acarbose-NCA on α-glucosidase (NCA, aqueous extract of *N. nucifera* combined with acarbose, fraction; the default effect). (b) Dose reduction index of nonconstant combo acarbose-NCA (NCA, aqueous extract of *N. nucifera* combined with acarbose, fraction; the default effect).

**Figure 18:**
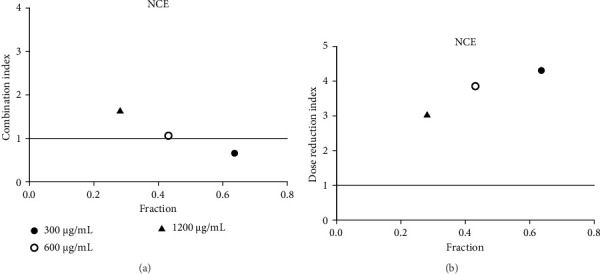
(a) Combination index plot of nonconstant combo acarbose-NCE on α-glucosidase (NCE, ethanolic extract of *N. nucifera* combined with acarbose, fraction; the default effect). (b) Dose reduction index of nonconstant combo acarbose-NCE (NCE, ethanolic extract of *N. nucifera* combined with acarbose, fraction; the default effect).

**Figure 19:**
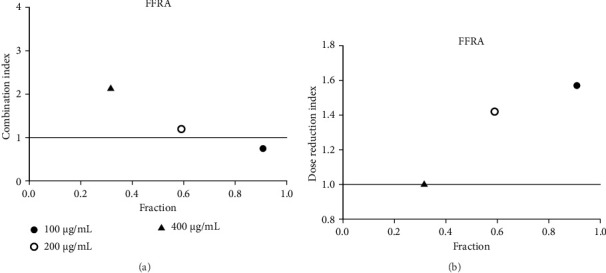
(a) Combination index plot of nonconstant combo acarbose-FFRA on α-glucosidase (FFRA, aqueous extract of five flower remedy combined with acarbose, fraction; the default effect). (b) Dose reduction index of nonconstant combo acarbose-FFRA (FFRA, aqueous extract of five flower remedy combined with acarbose, fraction; the default effect).

**Figure 20:**
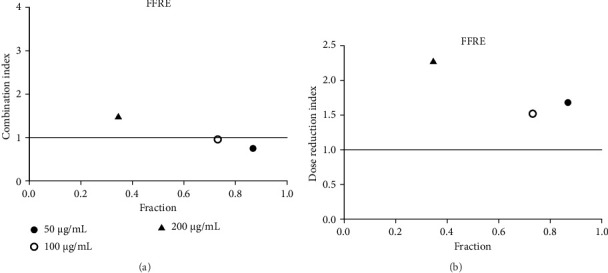
(a) Combination index plot of nonconstant combo acarbose-FFRA on α-glucosidase (FFRA, aqueous extract of five flower remedy combined with acarbose, fraction; the default effect). (b) Dose reduction index of nonconstant combo acarbose-FFRA (FFRA, aqueous extract of five flower remedy combined with acarbose, fraction; the default effect).

Acarbose was used in the CI analysis to evaluate the α-glucosidase inhibitory activity of 12 extracts. A fixed acarbose dose at its IC_50_ (170 μg/mL) was combined with varying concentrations of each extract, ranging from 0.5 to 2 times their respective IC_50_ values. The CI results are presented in Tables 7, 8, 9, 10, 11, 12, 13, 14, 15, 16, 17, and 18 and Figures 9, 10, 11, 12, 13, 14, 15, 16, 17, 18, 19, and 20. Most extracts showed synergistic effects when combined with acarbose at lower doses, except for JSA and MF extracts, which exhibited antagonistic interactions. JSA, particularly at lower concentrations (400 μg/mL), showed synergism and potential for dose reduction when combined with another drug (Figure 9). However, at higher concentrations (800 μg/mL and 1600 μg/mL), the effects shifted toward antagonism, making dose reduction less effective. At 1600 μg/mL, the data even suggested a significant negative impact on dose reduction. MS, especially at lower concentrations, demonstrated synergistic effects that could allow for dose reduction when combined with other drugs (Figure 15). However, at a higher concentration of MSA (300 μg/mL), the effects turned antagonistic, making dose reduction unlikely. For FFR extracts, both (FFRA and FFRE) showed synergistic effects at lower doses and had a dose reduction index greater than 1, indicating that the dosage of at least one drug could be reduced when used in combination.

### 3.8. Assessment of the Enzyme Kinetic Study of FFR on α-Amylase Inhibitory Activity

**Figure 21:**
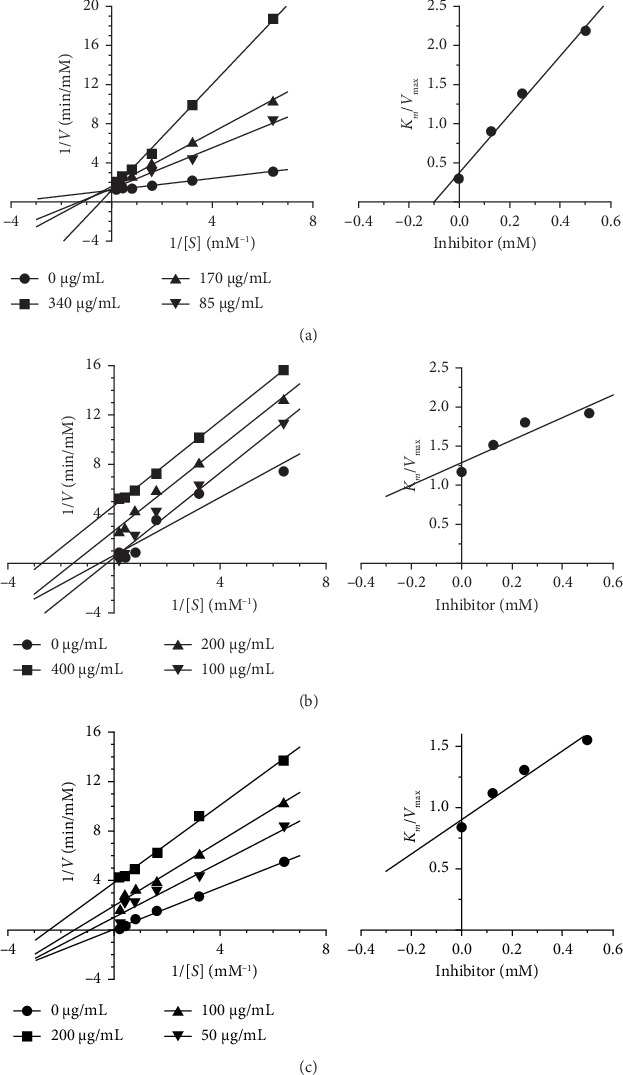
The mode of action of the standard drug, acarbose (a), and FFRA (b) and FFRE (c) extracts on α-amylase inhibitory activity.

Given the FFR extract's historic therapeutic use, the enzyme kinetics investigation sought to discover its kind of inhibition. Standard analytical techniques, such as the Lineweaver–Burk and Dixon methods, were employed to identify its mode of action. The Lineweaver–Burk plot showed that FFRA functioned as a noncompetitive inhibitor with a *K*_*i*_ value of 1.347 mM. Conversely, FFRE revealed competitive inhibition and had a *K*_*i*_ value of 0.274 mM. Acarbose, a well-known inhibitor, also demonstrated competitive inhibition, with a *K*_*i*_ value of 0.683 mM (Table 19 and Figure 21).

### 3.9. Assessment of the Enzyme Kinetic Study of FFR on α-Glucosidase Inhibitory Activity

**Figure 22:**
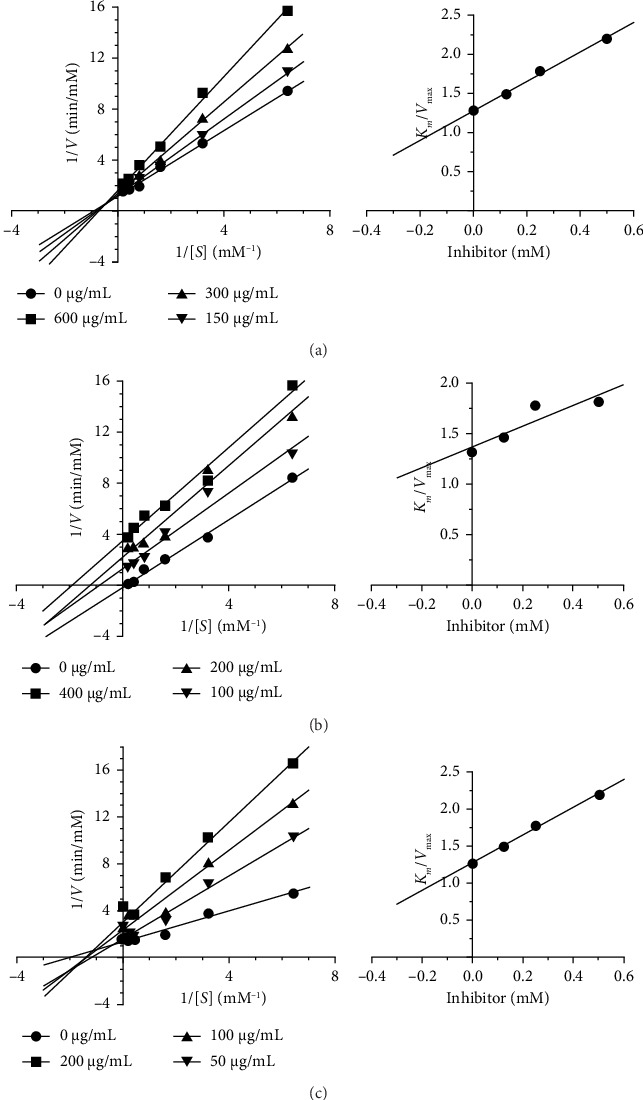
The mode of action of the standard drug, acarbose (a), and FFRA (b) and FFRE (c) extracts on α-glucosidase inhibitory activity.

Table 20 and Figure 22 show the kinetic characteristics of α-glucosidase after inhibition with FFR extracts and acarbose, a conventional inhibitor. Acarbose, as a conventional inhibitor, exhibits the highest inhibition (lowest *K*_*i*_, 0.102 mM) and acts on a competitive inhibition mechanism. FFRA has a higher *K*_*i*_ (0.891 mM) and shows mixed inhibition, indicating that it interferes with enzyme activity in multiple ways. FFRE has a modest *K*_*i*_ (0.645 mM) and exhibits noncompetitive inhibition, suggesting that it binds to a location other than the active site, which leads to enzyme inactivation.

## 4. Discussion

This study investigated into the potential of Thai traditional medicine, FFR, for managing diabetes. The study investigated how FFR inhibits the enzymes α-amylase and α-glucosidase, which break down carbohydrates into glucose. The objective was to determine whether the combination of flowers in FFR was more effective than individual flowers alone and to investigate how FFR could be used to lower blood sugar. The findings demonstrated that FFR strongly inhibited both enzymes and was more effective than any single flower extract, indicating that the flowers perform together synergically. It indicates that FFR could be an effective new treatment for diabetes. FFR inhibits these enzymes, delaying carbohydrate digestion and absorption, which assists in reducing blood sugar increases after meals, and is a key component in type 2 diabetes management [20]. α-Amylase, secreted in the saliva and pancreas, hydrolyzes complex carbohydrates into oligosaccharides [21], while α-glucosidase, located in the small intestine brush border, further breaks down oligosaccharides into glucose [22]. Inhibiting these enzymes delays carbohydrate digestion and absorption, thereby attenuating the postprandial glucose surge [23].

In this study, FFRE demonstrated the most potent inhibition of carbohydrate-digesting enzymes. Specifically, it displayed a superior ability to inhibit α-amylase, with an IC_50_ value of 145.05 μg/mL, which is significantly lower than that of both the individual flower extracts and the standard drug acarbose, which exhibited an IC_50_ value of 338.00 μg/mL. Furthermore, FFRE also exhibited the strongest inhibitory effect against α-glucosidase, with an IC_50_ value of 104.99 μg/mL, which is also significantly lower than that of the standard drug acarbose, which exhibited an IC_50_ value of 166.66 μg/mL. This supports the idea that traditional multiherb remedies can be highly effective [24]. Herbal remedies, with their complex mix of active compounds, may offer a wider range of therapeutic effects through multiple targets [25]. There is a common belief that herbal treatments are safer than pharmaceutical drugs, and while this is not universally true, some herbal remedies may indeed have fewer side effects [26]. This multitarget approach may be beneficial for complex diseases [11]. Many people believe that herbal remedies are safer and have fewer side effects than conventional drugs [27]. While this is not always true, some herbal remedies may have a lower risk of adverse effects compared to certain pharmaceuticals [28]. Ethanol's intermediate polarity allows it to extract a broad spectrum of bioactive compounds, including both polar (like flavonoids and phenolic acids) and some less polar substances. This versatility is a significant advantage [29]. Many valuable plant-derived compounds fall within this polarity range, making ethanol an effective extraction solvent. Compared to many other organic solvents (like methanol or hexane), ethanol is relatively safe for human consumption, especially when used in appropriate concentrations [30]. This is crucial for applications in food, pharmaceuticals, and cosmetics, where residual solvent levels must be strictly controlled [30]. This suggests that the combined effects of multiple bioactive compounds present in the FFR contribute to a more comprehensive and effective inhibition of carbohydrate-hydrolyzing enzymes. The observed inhibition levels are comparable to or even exceed those reported for some standard inhibitors, indicating the therapeutic potential of FFR.

A key finding of this study is the demonstration of synergistic interactions among the constituent flowers of FFR. CI is a crucial tool for quantifying and characterizing the interactions between multiple drugs, including herbal drugs. It provides a numerical value that indicates whether the combined effect is synergistic, additive, or antagonistic [26]. The CI is used to determine the nature and extent of drug interactions, particularly when assessing the efficacy of drug combinations [16]. The observed synergistic interactions, particularly in FFE and its plant ingredients, suggest that the combined effects of these herbal components are greater than the sum of their individual effects. This finding is significant for several reasons. Firstly, it supports the traditional use of polyherbal formulations, where the combined therapeutic effects are often attributed to complex interactions between various phytochemicals. Secondly, it highlights the potential for optimizing herbal therapies by strategically combining specific constituents to achieve enhanced efficacy at lower individual doses, potentially minimizing adverse effects [11]. Analysis of the combination effects revealed a trend towards synergistic inhibition of α-amylase and α-glucosidase at lower concentrations for the majority of individual flower extracts and the FFR. When examining the inhibition of α-amylase, a clear and distinct departure from the observed trends was seen with the FFRA. When combined with the standard inhibitor acarbose, the interaction consistently exhibited an antagonistic effect across the entire range of tested dosages, indicating that the combined effect was less than that of the individual components [31]. The investigation into the combined inhibitory activity of acarbose with FFR and its constituent plant ingredients on α-glucosidase yielded results consistent with those observed for α-amylase. Specifically, the combinations involving JSA, MFA, and MFE demonstrated antagonistic interactions, as indicated by CI values exceeding 1. To fully understand the synergistic effects, we observed, we need to explore several potential mechanisms. For example, it is possible that a small dose of FFRE increases the absorption or availability of another active ingredient. Another possibility is that the combined compounds act on multiple targets within the disease process, or that they cooperatively modify different parts of a shared pathway [26]. To validate these synergistic effects and determine the exact mechanisms involved, we recommend conducting pharmacokinetic studies to track compound movement, *in vitro* assays to examine their actions at a cellular level, and *in vivo* trials to assess their effectiveness in living systems. In contrast to synergistic effects, we observed antagonistic interactions when FFRA was used, specifically in its inhibition of the α-amylase enzyme. The CI values, consistently above 1 across all concentrations, indicate that these combinations actually reduce the intended therapeutic effect [32]. This reduction could stem from several factors, including competition between components for binding to the enzyme, interference with metabolic processes, or opposing pharmacological actions [33]. Therefore, it is essential to identify and avoid such antagonistic combinations in herbal formulations to prevent diminished efficacy.

Herbal extracts contain a multitude of compounds, each with the potential to interact with various biological targets, including enzymes [26]. This polypharmacological nature makes it challenging to pinpoint the exact mechanisms of inhibition. Therefore, when studying an herbal drug, it is possible that multiple compounds within that drug, could be acting as different types of enzyme inhibitors [34]. The study revealed that FFRA and FFRE inhibit α-amylase and α-glucosidase through distinct mechanisms. Specifically, FFRA acted as a noncompetitive inhibitor of α-amylase with a *K*_*i*_ value of 1.347 mM, noncompetitive inhibition occurs when an herbal compound binds to the enzyme-substrate complex, preventing the reaction from proceeding. This type of inhibition is less common but can occur with herbal constituents that interact with allosteric sites on the enzyme–substrate complex. His type of inhibition is more likely to occur in multisubstrate enzyme reactions. While FFRE acted as a competitive inhibitor with a *K*_*i*_ value of 0.274 mM, acarbose, a standard competitive inhibitor, had a *K*_*i*_ value of 0.683 mM. In competitive inhibition, an herbal compound competes with the substrate for binding to the enzyme's active site [35]. This type of inhibition can be relevant in herbal medicine, where certain phytochemicals may structurally resemble endogenous substrates. For example, some flavonoids or phenolic compounds might compete with endogenous molecules for binding to enzymes involved in inflammation or metabolism [36]. Notably, FFRA's higher *K*_*i*_ value compared to acarbose indicates a weaker binding affinity to the α-amylase enzyme [37]. FFRA and FFRE showed distinct inhibition patterns against α-glucosidase. Specifically, FFRA acted as a mixed inhibitor, with a *K*_*i*_ of 0.891 mM. This type of inhibition occurs when a compound binds to both the enzyme itself and the enzyme–substrate complex, but with varying strengths. This dual binding action reduces both the enzyme's activity and its ability to bind the substrate. Given the complex nature of herbal extracts, where numerous phytochemicals with diverse structures are present, mixed inhibition is likely a frequent mechanism [38]. Conversely, FFRE exhibited noncompetitive inhibition, with a *K*_*i*_ of 0.645 mM. For comparison, acarbose, a known competitive inhibitor, had a *K*_*i*_ value of 0.102 mM.

While our findings demonstrate the promising antidiabetic potential of the FFR and its individual extracts through their inhibitory effects on α-amylase and α-glucosidase, the precise molecular entities responsible for this activity remain undefined. To fully elucidate the therapeutic mechanisms and pave the way for potential drug development, a comprehensive phytochemical investigation is imperative.

Specifically, future research must prioritize the identification and quantification of the individual bioactive compounds within the FFR extracts. This necessitates the application of advanced analytical techniques, notably high-performance liquid chromatography (HPLC) coupled with mass spectrometry (MS). HPLC, with its ability to separate complex mixtures based on their physicochemical properties, will enable the isolation of individual components within the FFR extracts. Subsequently, MS, particularly techniques like tandem mass spectrometry (MS/MS), will provide detailed structural information, allowing for the unambiguous identification of these compounds. This combined approach will generate a comprehensive chemical profile of the FFR extracts, revealing the array of constituents present and their relative abundances.

Beyond mere identification, it is crucial to establish a direct correlation between specific phytochemicals and the observed antidiabetic activity. This can be achieved through bioactivity-guided fractionation, where individual compounds or fractions are tested for their inhibitory effects on α-amylase and α-glucosidase. By systematically eliminating inactive compounds, the key contributors to the antidiabetic potential of FFR can be pinpointed.

Furthermore, computational approaches, specifically molecular docking studies, will be instrumental in understanding the molecular interactions between the identified bioactive compounds and the target enzymes. Molecular docking simulations can predict the binding modes and affinities of these compounds within the enzyme's active site, providing insights into the mechanisms of inhibition. These simulations will not only validate the experimental findings but also identify key residues involved in the enzyme-inhibitor interactions. Understanding these interactions at the molecular level is crucial for optimizing the efficacy of FFR-derived compounds and for designing novel antidiabetic agents.

In summary, a multifaceted approach combining advanced analytical techniques, bioactivity-guided fractionation, and molecular docking studies is essential for a thorough understanding of the antidiabetic mechanisms of FFR. This comprehensive investigation will not only identify the active principles but also provide a strong foundation for future preclinical and clinical studies, ultimately leading to the development of effective and safe herbal-based antidiabetic therapies.

## 5. Conclusions

This study explored the antidiabetic potential of FFR, a Thai traditional medicine, by evaluating its inhibitory effects on α-amylase and α-glucosidase, examining synergistic interactions, and investigating potential mechanisms of action. Our findings revealed significant inhibition of both enzymes by FFR. Notably, the combination of FFRA and acarbose demonstrated a remarkable synergistic effect on α-glucosidase inhibition at low doses. Furthermore, FFRE exhibited greater potency in inhibiting α-amylase and α-glucosidase than the standard drug acarbose, acting through competitive and noncompetitive mechanisms, respectively. These results offer valuable insights into FFR's pharmacological properties and contribute to the understanding of traditional medicine. Future research should focus on identifying the active compounds within FFR, conducting *in vivo* studies, and ultimately performing clinical trials.

## Figures and Tables

**Table 1:** List of plant species and the specific parts utilized in the study.

No	Plant species	Family	Plant part	Voucher number
1	*Jasminum sambac* Ait.	Oleaceae	Flowers	SMD 191 003035
2	*Mimusops elengi* L.	Sapotaceae	Flowers	SMD 042 004001
3	*Mesua ferrea* Linn.	Guttiferae	Flowers	SMD 261 002011
4	*Mammea siamensis* Kosterm.	Guttiferae	Flowers	SMD 122 007001
5	*Nelumbo nucifera* Gaertn.	Nelumbonaceae	Stamens	SMD 181 001001

**Table 2:** Extraction yields of ethanolic and aqueous extracts from medicinal plants in FFR.

Plant species	Extraction yield (%w/w)	
	Ethanolic extract	Aqueous extract
*Jasminum sambac* Ait.	2.85	7.45
*Mimusops elengi* L.	14.54	21.24
*Mesua ferrea* Linn.	5.62	15.87
*Mammea siamensis* Kosterm.	11.47	24.36
*Nelumbo nucifera* Gaertn.	6.12	12.65
FFR	17.78	10.72

**Table 3:** Combination index (CI) on α-amylase inhibitory activity of standard drug (acarbose) and MEA.

Name	Sample; MEA				
Standard; acarbose	Concentration (μg/mL)		150	300	600
		% Inhibition (CI)^∗^	36.57	44.30	58.69
	300	35.28	52.03 (0.49)^∗^	72.03 (1.04)^∗^	87.63 (3.83)^∗^

^∗^CI = Combination index.

**Table 4:** Combination index (CI) on α-amylase inhibitory activity of standard drug (acarbose) and MFA.

Name	Sample; MFA				
Standard; acarbose	Concentration (μg/mL)		75	150	300
		% Inhibition (CI)^∗^	35.97	58.14	79.13
	300	35.28	72.26 (0.69)^∗^	92.70 (1.42)^∗^	94.38 (3.46)^∗^

^∗^CI = Combination index.

**Table 5:** Combination index (CI) on α-amylase inhibitory activity of standard drug (acarbose) and FFRA.

Name	Sample; FFRA				
Standard; acarbose	Concentration (μg/mL)		75	150	300
		% Inhibition (CI)^∗^	46.31	70.95	83.43
	300	35.28	94.13 (1.03)^∗^	96.34 (1.96)^∗^	97.65 (3.78)^∗^

^∗^CI = Combination index.

**Table 6:** Combination index (CI) on α-amylase inhibitory activity of standard drug (acarbose) and FFRE.

Name	Sample; FFRE				
Standard; acarbose	Concentration (μg/mL)		75	150	300
		% Inhibition (CI)^∗^	40.44	49.43	76.06
	300	35.28	94.37 (0.64)^∗^	97.09 (1.94)^∗^	98.43 (5.44)^∗^

^∗^CI = Combination index.

**Table 7:** Combination index (CI) on α-glucosidase inhibitory activity of standard drug (acarbose) and JSA.

Name	Sample; JSA				
Standard; acarbose	Concentration (μg/mL)		400	800	1600
		% Inhibition (CI)^∗^	41.21	59.98	76.53
	170	46.87	77.21 (1.22)^∗^	87.84 (2.26)^∗^	94.31 (4.22)^∗^

^∗^CI = Combination index.

**Table 8:** Combination index (CI) on α-glucosidase inhibitory activity of standard drug (acarbose) and JSE.

Name	Sample; JSE				
Standard; acarbose	Concentration (μg/mL)		500	1000	2000
		% Inhibition (CI)^∗^	43.56	56.89	68.89
	170	46.87	45.58 (0.67)^∗^	57.31 (0.95)^∗^	69.71 (1.87)^∗^

^∗^CI = Combination index.

**Table 9:** Combination index (CI) on α-glucosidase inhibitory activity of standard drug (acarbose) and MEA.

Name	Sample; MEA				
Standard; acarbose	Concentration (μg/mL)		200	400	800
		% Inhibition (CI)^∗^	25.02	59.65	75.80
	170	46.87	61.02 (0.82)^∗^	71.65 (1.32)^∗^	81.80 (2.14)^∗^

^∗^CI = Combination index.

**Table 10:** Combination index (CI) on α-glucosidase inhibitory activity of standard drug (acarbose) and MEE.

Name	Sample; MEE				
Standard; acarbose	Concentration (μg/mL)		150	300	600
		% Inhibition (CI)^∗^	43.71	48.96	53.02
	170	46.87	73.72 (0.85)^∗^	84.35 (1.50)^∗^	92.86 (3.02)^∗^

^∗^CI = Combination index.

**Table 11:** Combination index (CI) on α-glucosidase inhibitory activity of standard drug (acarbose) and MFA.

Name	Sample; MFA				
Standard; acarbose	Concentration (μg/mL)		75	150	300
		% Inhibition (CI)^∗^	25.27	51.70	69.90
	170	46.87	44.48 (5.17)^∗^	63.70 (6.74)^∗^	81.90 (10.83)^∗^

^∗^CI = Combination index.

**Table 12:** Combination index (CI) on α-glucosidase inhibitory activity of standard drug (acarbose) and MFE.

Name	Sample; MFE				
Standard; acarbose	Concentration (μg/mL)		75	150	300
		% Inhibition (CI)^∗^	51.31	61.72	73.72
	170	46.87	54.87 (5.22)^∗^	63.95 (6.81)^∗^	79.72 (10.91)^∗^

^∗^CI = Combination index.

**Table 13:** Combination index (CI) on α-glucosidase inhibitory activity of standard drug (acarbose) and MSA.

Name	Sample; MSA				
Standard; acarbose	Concentration (μg/mL)		75	150	300
		% Inhibition (CI)^∗^	51.86	69.05	78.50
	170	46.87	75.86 (0.84)^∗^	87.06 (1.83)^∗^	90.16 (2.44)^∗^

^∗^CI = Combination index.

**Table 14:** Combination index (CI) on α-glucosidase inhibitory activity of standard drug (acarbose) and MSE.

Name	Sample; MSE				
Standard; acarbose	Concentration (μg/mL)		200	400	800
		% Inhibition (CI)^∗^	49.56	63.61	80.89
	170	46.87	55.56 (0.92)^∗^	69.31 (1.31)^∗^	88.77 (1.94)^∗^

^∗^CI = Combination index.

**Table 15:** Combination index (CI) on α-glucosidase inhibitory activity of standard drug (acarbose) and NCA.

Name	Sample; NCA				
Standard; acarbose	Concentration (μg/mL)		200	400	800
		% Inhibition (CI)^∗^	26.04	51.70	75.05
	170	46.87	62.60 (0.79)^∗^	75.70 (1.37)^∗^	99.05 (15.04)^∗^

^∗^CI = Combination index.

**Table 16:** Combination index (CI) on α-glucosidase inhibitory activity of standard drug (acarbose) and NCE.

Name	Sample; NCE				
Standard; acarbose	Concentration (μg/mL)		300	600	1200
		% Inhibition (CI)^∗^	43.82	62.11	71.14
	170	46.87	61.02 (0.66)^∗^	74.24 (1.07)^∗^	83.60 (1.66)^∗^

^∗^CI = Combination index.

**Table 17:** Combination index (CI) on α-glucosidase inhibitory activity of standard drug (acarbose) and FFRA.

Name	Sample; FFRA				
Standard; acarbose	Concentration (μg/mL)		100	200	400
		% Inhibition (CI)^∗^	40.87	60.54	74.59
	170	46.87	45.86 (0.75)^∗^	64.56 (1.20)^∗^	80.97 (2.16)^∗^

^∗^CI = Combination index.

**Table 18:** Combination index (CI) on α-glucosidase inhibitory activity of standard drug (acarbose) and FFRE.

Name	Sample; FFRE				
Standard; acarbose	Concentration (μg/mL)		50	100	200
		% Inhibition (CI)^∗^	43.83	62.16	68.99
	170	46.87	47.90 (0.91)^∗^	66.16 (1.26)^∗^	79.25 (1.86)^∗^

^∗^CI = Combination index.

**Table 19:** The presented data showcases the kinetic parameters of α-amylase when exposed to the extract of FFR and acarbose (standard drug).

Inhibitors	α-Amylase	
	*K*_*i*_ (mM)	Mode
Acarbose	0.683	Competitive
FFRA	1.347	Noncompetitive
FFRE	0.274	Competitive

**Table 20:** The presented data showcases the kinetic parameters of α-glucosidase when exposed to the extract of FFR and acarbose (standard drug).

Inhibitors	α-Glucosidase	
	*K*_*i*_ (mM)	Mode
Acarbose	0.102	Competitive
FFRA	0.891	Mixed
FFRE	0.645	Noncompetitive

## Data Availability

The data that support the findings of this study are available upon request from the corresponding author.

## Nomenclature

DM: Diabetes mellitus
FFR: Five flower remedy
FFRE: Ethanolic extract of five flower remedy
FFRA: Aqueous extract of five flower remedy
JS: *Jasminum sambac* Ait.
ME: *Mimusops elengi* L.
MF: *Mesua ferrea* Linn.
MS: *Mammea siamensis* Kosterm.
NC: *Nelumbo nucifera* Gaertn
DMSO: Dimethyl sulfoxide
Na_2_CO_3_: Sodium carbonate
PBS: Phosphate-buffered saline
*K*_*i*_: Inhibition constant
*CNP*: 2-chloro-4-nitrophenol
*CNPG3*: 2-chloro-4-nitrophenyl-α-D-maltotrioside
*pNP*: Para-nitrophenyl-α-glucopyranoside
*pNPG*: Para-nitrophenyl-α-D-glucopyranoside
IC_50_: Half-maximal inhibitory concentration
RIIE: Research and Innovation Institute Excellence
